# 3C3R, an Image Encryption Algorithm Based on BBI, 2D-CA, and SM-DNA

**DOI:** 10.3390/e21111075

**Published:** 2019-11-02

**Authors:** Sajid Khan, Lansheng Han, Ghulam Mudassir, Bachira Guehguih, Hidayat Ullah

**Affiliations:** 1 School of Computer Science and Technology, Huazhong University of Science and Technology, Wuhan 430074, China; i201722242@hust.edu.cn; 2 Faculty of Computer Science Department, Huazhong University of Science and Technology, Wuhan 430074, China; 3 School of Information and Communication Technologies, University of L’Aquila, 67100 L’Aquila, Italy; ghulam.mudassir@graduate.univaq.it; 4 School of Communication and Information Engineering, Shanghai University, Shanghai 200444, China; hidayat@shu.edu.cn

**Keywords:** image encryption, DNA sequence, State Machine, bit inversion, entropy, cellular automata, security

## Abstract

Color image encryption has enticed a lot of attention in recent years. Many authors proposed a chaotic system-based encryption algorithms for that purpose. However, due to the shortcomings of the low dimensional chaotic systems, similar rule structure for RGB channels, and the small keyspace, many of those were cryptanalyzed by chosen-plaintext or other well-known attacks. A Security vulnerability exists because of the same method being applied over the RGB channels. This paper aims to introduce a new three-channel three rules (3C3R) image encryption algorithm along with two novel mathematical models for DNA rule generator and bit inversion. A different rule structure was applied in the different RGB-channels. In the R-channel, a novel Block-based Bit Inversion (**BBI**) is introduced, in the G-channel Von-Neumann (VN) and Rotated Von-Neumann (RVN)- based 2D-cellular structure is applied. In the B-channel, a novel bidirectional State Machine-based DNA rule generator (**SM-DNA**) is introduced. Simulations and results show that the proposed **3C3R** encryption algorithm is robust against all well-known attacks particularly for the known-plaintext attacks, statistical attacks, brute-force attacks, differential attacks, and occlusion attacks, etc. Also, unlike earlier encryption algorithms, the **3C3R** has no security vulnerability.

## 1. Introduction

With the vast improvement of network and communication technology, communications were improved significantly. Communication for multimedia content, especially the image content over the Internet has become more and more chronic. Nevertheless, the security of the multimedia has a serious risk in the progression of communication because of the openness and distribution of the Internet. So the public has to take great care in terms of confidentiality and security of multimedia communication [1].

Among several protecting approaches, image encryption is one of the most efficient and frequent strategies for digital information protection. Now, greater and more new techniques are proposed for image encryption, which pursuits to reduce image content’s redundancy by means of distinctive operations, such as DNA-based encryption operations [2] and chaos-based ciphers [3,4]. Many solo image encryption schemes were proposed by researchers to address the multiple-image encryption algorithms. The most widely used image encryption algorithms encompass chaotic-based image encryption [5,6,7,8,9], image encryption in the transform domain [10,11,12,13], DNA-based image encryption [14,15], and evolutionary-based image encryption [16].

The chaos system possesses a ramification of traits, which includes high sensitivity to initial conditions, determinacy, and ergodicity. Sequences produced by chaotic maps are habitually pseudo-random sequences, and their structures are very complicated and tough to be analyzed and foretold [17,18,19]. The typical ciphers primarily based on a chaotic map may be partitioned into two degrees: diffusion and permutation. In exercise, researchers frequently combine permutation and diffusion to get more computational security.

Because of the sensitivity and pseudo-randomness of the chaos, the chaotic sequences generated with the help of chaotic systems are intermittent and complicated. Researchers mostly encrypt the image in an early stage through the chaotic structures obtained by the low dimensional chaotic system, such as the one introduced in [20]. An image encryption technique with a new 1D chaotic system was proposed.

In [21] the author introduced 2D Logistic-Sine chaotic map-based image encryption algorithm. However, these sequences generated through low dimensional chaotic structures, and due to the inadequacy of the small keyspace cannot withstand against brute force attacks and thus possess the low security.

Conversely, Hyper-chaotic systems own larger keyspace and extra complicated dynamic features. Thus, the hyper-chaotic-based system can compensate for the shortcomings of a small keyspace of the low dimensional chaotic system and are more appropriate for the image encryption. In [22,23] the authors proposed a hyper-chaotic systems and fuzzy cellular automata-based novel image encryption algorithm that has higher security.

Sajid et al. [24] introduced a hybrid image encryption algorithm based on FSM and cellular automata accompanying DNA sequence. The algorithm has good results and also the concept of a local rule is appreciable with regards to algorithm efficiency. However, the shortcomings of the particular algorithm is its aimed for use only for grayscale images.

Huang et al. [25] proposed 7 dimensional CNN hyper-chaos-based application of image encryption scheme. Based on the results, the authors claimed that the hyper-chaotic systems are better than the low dimensional chaotic systems that own small keyspace. Astonishing information density, and the massive parallelism characteristics of DNA sequences, pushed the researchers to introduce collective hyper-chaos and DNA methods. Such collective technology schemes can be proved to be highly efficient and secure multimedia encryption schemes [26,27,28].

A new encryption algorithm for the color image was proposed in [29] that employs the hash-256 function to amend the control parameters and initial values of the chaotic system. The red, green and the blue channels of the image arranged into vector array form of one-dimensional. Then by rendering to the chaotic sequence generated by Piecewise Linear Chaotic Map 1-D vector array get sorted.

Nonetheless, in the utmost chaotic-based image encryption schemes that were stated above, the permutation phase and diffusion phase are autonomous of the plain image. Because the cryptosystem is impervious to the plain images and secret keys, such structures have the security flaws and cannot resist chosen/known-plaintext attacks or differential attacks. Table 1, listed some well known encryption algorithms that get successfully Cryptanalysis by the enlisted attack approach.

Therefore, after the analysis of above-mentioned papers, we proposed a novel three channel three rule (3C3R) color image encryption algorithm. We made three contributions to this paper with each having corresponding merits.

Firstly, for the red channel: a new block bit inversion (BBI) model was proposed. In every row simultaneous right and left two-bits selection is random and relies upon the integer value of a particular pixel and the block multiplier. The left and right direction bit selection efficiently lessen the pixel correlation among the plain and ciphered image.

Secondly, for the green channel: cellular automata-based confined rules structure with Von-Neumann (VN) and Rotated Von-Neumann (RVN) structure was applied. Each sub-matrix gets different structural rule based on the particular bit values. Except for the fact that in the rule selection every sub-matrix has a direct relation with the previous one.

Thirdly, for the blue channel: a novel mathematical model of bidirectional State Machine (SM)-based DNA rule generator (SM-DNA) was proposed. The proposed model efficiently generates a random rule for each block-matrix. In each block-matrix $i^{th}$ and $j^{th}$ bit used as an input for the rule selection of next block-matrix. That means the bit arrangement of every predecessor block-matrix is responsible for the rule selection of the next block-matrix. The remainder of the paper organized as comply with; in Section 2: Literature survey/Preliminary work, Section 3: Proposed Image encryption scheme. Section 4: Experimental parameters and discussion while Section 5: Security analysis with the following Section 6: Conclusion and future work.

## 2. Literature Survey

A binary sequence $bin_{\left(n-1\right)},bin_{\left(n-2\right)},\ldots ,bin_{\left(1\right)},bin_{\left(0\right)}$ can be used to denote a non-negative decimal number (DN) by the following equation.

(1) $$DN=\sum_{i=0}^{n-1}bin_{\left(i\right)}2^{i}=bin_{\left(0\right)}2^{0}+bin_{\left(1\right)}2^{1}+\ldots +bin_{\left(n-1\right)}2^{n-1}$$

As in any image, the pixel values of each channel are the non-negative decimal numbers between 0 and 255, thus every pixel can be denoted by the binary sequence of 1-byte or 8-bits. Similarly, the whole image can also be decomposed into binary 8-bit-planes [50]. In such case, the $i^{th}$ bit-plane will comprise of all the $i^{th}$ bits of the binary demonstration of every pixel. Among these 2^3^ = 8 bit-planes, most left bit-plane contains the highest significant visual information of the plain image, while the right most bit-plane contains the least visual information.

### 2.1. Scrambling Method

**Figure 1:**
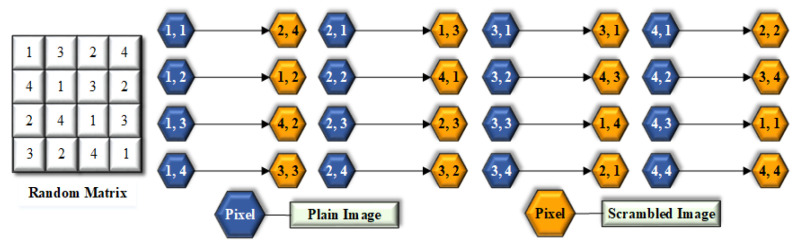
Scrambling one-to-one mapping.

Scrambling is an easy and effective technique to lessen the correlation between the neighboring pixels. This paper introduced a one-to-one mapping for scrambling. Due to simultaneous changes of pixels in columns and rows, the proficiency of the algorithm enhanced enormously concerning time. The proposed algorithm has a one-to-one ratio between the pixels of scrambled image and the plain image, as displayed in Figure 1.

The pseudo-code of the scrambling method for Encryption process is described in Algorithm 1, while in the decryption process the same procedure is applied but using reverse shifting, as described in Algorithm 2.

**Algorithm 1** Scrambling

**Encryption Process:**
**Input:** Generate S(i,j) matrix equal to the size of Plain image matrix P(M,N).**Output:**T(m,n) size Scrambled matrix. Suppose for **M** = 4 and **N** = 4;$S\in Z^{\left(M\times N\right)}$; $P\in Z^{\left(M\times N\right)}$; $T\in Z^{\left(M\times N\right)}$;*A* = Array sequence [3,2,1,4];*B* = Array sequence [4,2,1,3];*I* = A.index(i) for *i* in Sorted[A];*J* = B.index(j) for *j* in Sorted[B];
$P_{pixels}=\left[\begin{array}{cccc} 101 & 103 & 182 & 14\\ 83 & 201 & 90 & 20\\ 92 & 49 & 100 & 34\\ 223 & 92 & 140 & 10 \end{array}\right]$
img = np.zeros((4,4));img[0,:] = [101,103,182,14];img[1,:] = [83,201,90,20];img[2,:] = [92,49,100,34];img[3,:] = [223,92,140,10];**S** = np.zeros((M,N));**for***i***in range**(0,M); **do** **for** *j* **in range** (0,N); **do**  m = [(j−I(i)−1)modN]+1  S[i,m−1] = J[j]  S=S+1
  **S** **end for**
**end for**
T = np.zeros((4,4))**for***j***in range**(0,N); **do** data = [ ]; **for** *i* **in range** (0,M); **do**  data.append.img [I,S(I,j)−1]  a = shift [data,(N/2)−S(0,j)] **end for**
**end for**
**for***k***in range** 1 to *N*; **do** T[k,S[k,j]] = a[k];
**end for**
S = $\left[\begin{array}{cccc} 1 & 3 & 2 & 4\\ 4 & 1 & 3 & 2\\ 2 & 4 & 1 & 3\\ 3 & 2 & 4 & 1 \end{array}\right]$; $\vec{Scr.(T)}=\left[\begin{array}{cccc} 140 & 103 & 83 & 100\\ 34 & 223 & 90 & 101\\ 92 & 20 & 14 & 92\\ 201 & 182 & 49 & 10 \end{array}\right]$


**Algorithm 2** Scrambling

**Decryption Process:**
**Input:** Random matrix S(i,j) and Cipher image matrix C(M,N).**Output:** Descrambled matrix **P**.**for***j***in range**(0,N); **do** data = [ ]; **for** *i* **in range** (0,M); **do**  data.append.img [I,S(I,j)−1]  a = shift [data,(N/2)−S(0,j)] **end for**
**end for**
**for***k***in range** 1 to *N*; **do** P[k,S[k,j]]↦a[k];
**end for**



### 2.2. Novel Block Bit Inversion (BBI)

**Figure 2:**
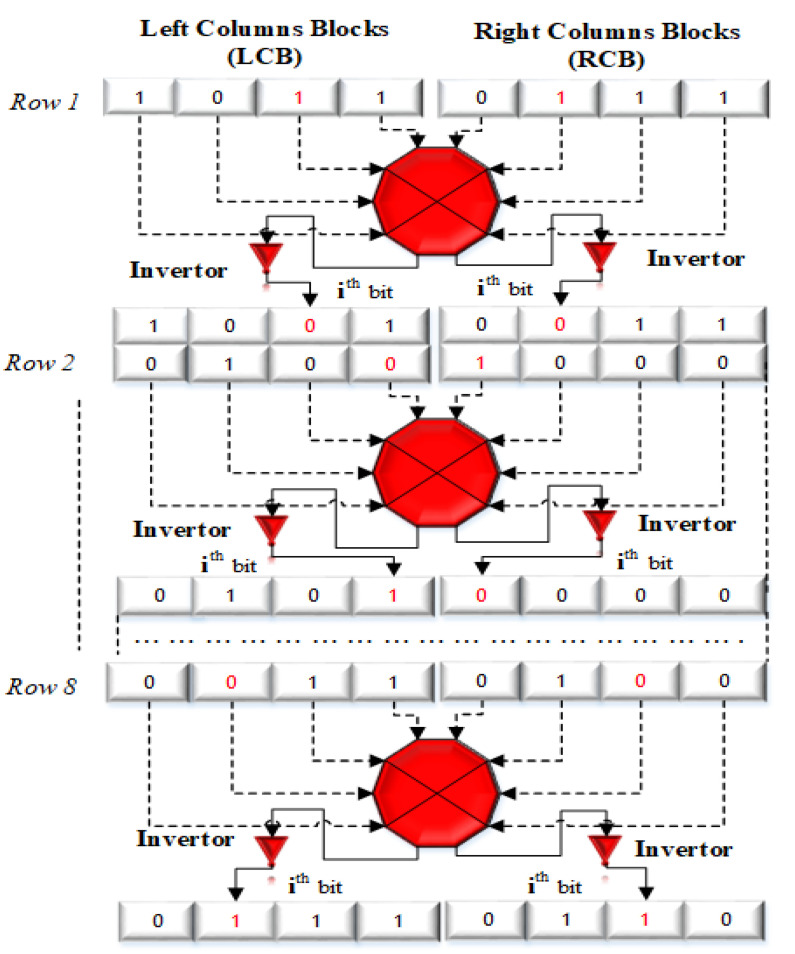
Block bit Inversion.

In this paper, we propose a novel blocked-based bit inversion technique to alter the pixel values. The Figure 2 is the graphical illustration of the proposed **BBI** structure for the image sub-matrix of the size M×N. Firstly, it divides the particular block matrix into two equal column blocks; labelled as Left bit Columns Blocks (LCB) and Right bit Columns Blocks (RCB). The purpose is to change the bits in every row and columns but in the opposite manner. It changes the particular bit **1** or **0** into opposite i.e., **0** if it is **1** or else **1** if it is **0**. For each pixel, the change in value occurs (either addition or subtraction) depending upon either its get converted into **1** from **0** or get converted to **0** from **1**. As each bit has a unique value, so because of bit inversion following change occurs in pixels based on the particular bit location.

$$MSB↔8^{th}bit\mapsto 2^{8-1}=2^{7}=\pm 128;\;7^{th}bit\mapsto 2^{8-2}=2^{6}=\pm 64$$

$$6^{th}bit\mapsto 2^{8-3}=2^{5}=\pm 32;\;5^{th}bit\mapsto 2^{8-4}=2^{4}=\pm 16$$

$$4^{th}bit\mapsto 2^{8-5}=2^{3}=\pm 8;\;3^{rd}bit\mapsto 2^{8-6}=2^{2}=\pm 4$$

$$2^{nd}bit\mapsto 2^{8-7}=2^{1}=\pm 2;\;LSB↔1^{st}bit\mapsto 2^{8-8}=2^{0}=\pm 1$$

**Figure 3:**
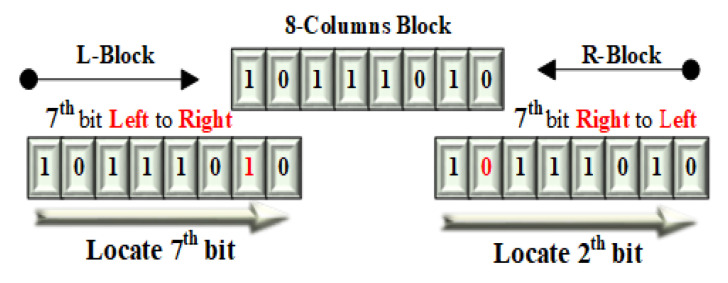
Bit selection.

For example, we can see in Figure 2 that for each binary blocks the bit selection is in opposite direction means $LCB_{bit}⇌RCB_{bit}$ for left and right columns blocks. For the left columns block the selection of bit is from left to right $LCB_{bit}⟹RCB_{bit}$ and for the right columns block the selection of bit is from right to left $LCB_{bit}⟸RCB_{bit}$. How its locate; is illustrated in Figure 3. Like in main diagram for the 1^*st*^ row, 3^*rd*^ bit is selected for the inversion in left columns block which is actually 6^*th*^ while counting from right to left. In same passion, for the right columns block, 3^*rd*^ bit is selected that is actually 6^*th*^ when counting from left to right. So selection is the same for 3^*rd*^ bit but direction is opposite as Left⇌Right. Thus, to select the particular bit in the novel BBI model, the formula efficiently locate the right and left block bits by proceeding only by the left to the right direction. The starting bit selection for each 8×8 blocks is based on the following formula.

(2) $$LCB_{bit}^{n}=\left[\left(n^{2}\times PmodB+1\right)+R_{i}\right]modB+1$$

Here i=1,2,3,…,8, where *P* is the pixel value of particular pixel, *n* is the block number multiplier to change the 1^*st*^ bit for each block, *B* is the byte (8 bits), mod is the mod function that returns the remainder between **0** to **7** because of B is one byte (8-bits), and $R_{i}$ is the particular row number range from **1** to **8**.

For better understanding, Let suppose the decimal value of **P** for the 1^*st*^ sub-matrix came **183**, so the value of left columns block ($LCB_{bit}$) for the **1^*st*^** row i=1 or $R_{i}$ = 1 can be gotten as follows.

$$LCB_{bit}^{n}=\left[\left(n^{2}\times PmodB+1\right)+R_{i}\right]modB+1$$

$$LCB_{bit}^{1}=\left[\left(1\times 183mod8+1\right)+1\right]mod8+1$$

$$\Rightarrow \left[7+1+1\right]mod8+1=1+1=2^{nd}bit$$

Likewise, for the 2^*nd*^ row of this 1^*st*^ sub-block matrix, i=2 or *R*_i_ = 2 the inverting bit will be;

$$LCB_{bit}^{1}=\left[\left(1\times 183mod8+1\right)+2\right]mod8+1$$

$$\Rightarrow \left[7+1+2\right]mod8+1=2+1=3^{rd}bit$$

Similarly, for the $3^{rd}$, $4^{th}$, $5^{th}$ to up to $8^{th}$ rows for every $LCB_{bit}^{n^{th}}$, desired inverting bit can be gotten through the formula. Alternatively, the formula of the bit selection for the right blocks is as follows.

(3) $$RCB_{bit}^{n}=\left[B-LCB_{bit}^{n}\right]modB+1$$

As an example, in the above case after putting values.

$$RCB_{bit}^{1}=\left[8-2\right]modB+1\Rightarrow 6mod8+1=7^{th}bit$$

Similarly, for the 2^*nd*^ row, the bit will be following.

$$RCB_{bit}^{1}=\left[8-3\right]modB+1\Rightarrow 5mod8+1=6^{th}bit$$

First of all, an initial configuration matrix of 8×8 is generated by the hash value of the key. Based on the above-mentioned formula of $RCB_{bit}$ and $LCB_{bit}$, bit inversion is done over the initial configuration matrix $\left(ICM_{i}^{t}\right)$ and later this updated initial configuration matrix $\left(ICM_{i}^{t+1}\right)$ is XORed with the first plain matrix. The general equation of XORing the updated $\left(ICM_{i}^{t+1}\right)$ with the plain image matrices is as follows.

(4) $$SM_{n(i,j)}^{t+1}=\left\{\begin{array}{cc} SM_{1}^{t}⊕ICM_{0}^{t+1} & n=1\\ SM_{n}^{t}⊕ICM_{n-1}^{t+1} & n>1 \end{array}\right.$$

where n=1,2,3,…,N; where *N* is the entire number of sub-matrices of the red channel. While $SM_{i}^{t+1}$ is the particular updated matrix, $SM_{i}^{t}$ is the plain image sub-matrix of the red channel, and $ICM_{i}^{t+1}$ is the updated initial configuration matrix. Similarly, for the 2^*nd*^ sub-matrix bit inversion is performed over the updated initial configuration matrix of 1^*st*^ block stage and then XORed with the second sub-matrix of the plain image as follows.

(5) $$SM_{2}^{t+1}=SM_{2}^{t}⊕ICM_{1}^{t+1}$$

For the decryption phase, the reverse process to get back the plain image matrix is as follows.

(6) $$SM_{n(i,j)}^{t}=\left\{\begin{array}{cc} SM_{1}^{t+1}⊕ICM_{0}^{t+1} & n=1\\ SM_{n}^{t+1}⊕ICM_{n-1}^{t+1} & n>1 \end{array}\right.$$

where n=1,2,3,⋯,N and *N* is the total number of sub-matrices.

### 2.3. Two-Dimensional Cellular Automata

**Figure 4:**
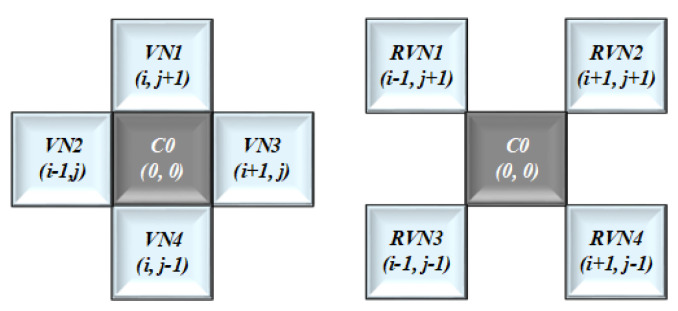
Von-Neumann (VN) + Rotated VN.

In this paper, a 2D-CA with VN and RVN structure was implemented as shown in Figure 4. Also, in cellular matrix form, every cell can have one of the two possible states that is either **1** or **0**. In cellular representation every cell has its neighboring cells. The cell with VN neighbors or a radius equivalent to **1** can be represented in equation form as follow:

(7) $$C_{vn}^{t+1}=\delta \left(C_{i,j+1}^{t}:C_{i-1,j}^{t}:C_{i,j}^{t}:C_{i+1,j}^{t}:C_{i,j-1}^{t}\right)$$

In this Equation (7), δ is the Boolean function or a state transition function that takes to the new state, where δ:C×∑↦ρ(C). Like VN, the cell with Rotated VN neighbors can be represented as follow.

(8) $$C_{rvn}^{t+1}=\delta \left(C_{i-1,j+1}^{t}:C_{i+1,j+1}^{t}:C_{i,j}^{t}:C_{i-1,j-1}^{t}:C_{i+1,j-1}^{t}\right)$$

So, to get the combined structure of VN and RVN, We merged these two equations as follows.

(9) $$\begin{array}{c} C_{M(i,j)}^{t+1}=\delta \left(C_{i-1,j+1}^{t}:C_{i+1,j+1}^{t}:C_{i+1,j}^{t}:C_{i,j+1}^{t}:C_{i,j}^{t}:C_{i-1,j+1}:C_{i-1,j-1}:C_{i+1,j-1}:C_{i+1,j+1}\right) \end{array}$$

We represent these **9** state variable with $C_{NW}:C_{N}:C_{NE}:C_{W}:C_{0}$: $C_{E}:C_{SW}:C_{S}:C_{SE}$, as shown in Table 2. For the green channel as the bit depth or intensity is **2^8^** so, we mapped the above mentioned **8** state variables with these eight bits as $C_{NW};C_{N};C_{NE};C_{W};C_{E}C_{SW};C_{S};C_{SE}$ = [1;1;1;1;1;1;1;1], while excluding the central state variable $C_{0(0,0)}^{t}$ represented as $C_{0}$.

Finally, the confined rule structure is based on the following equation.

(10) $$\begin{array}{c} C_{M(i,j)}^{t+1}=\left(C_{NW}\times C_{i-1,j+1}^{t}\right)⊕\left(C_{N}\times C_{i,j+1}^{t}\right)⊕\left(C_{NE}\times C_{i+1,j+1}^{t}\right)⊕\left(C_{E}\times C_{i-1,j}^{t}\right)⊕\left(C_{3}\times C_{i+1,j}^{t}\right)⊕\\ \left(C_{SW}\times C_{i-1,j-1}\right)⊕\left(C_{S}\times C_{i,j-1}\right)⊕\left(C_{SE}\times C_{i+1,j-1}\right) \end{array}$$

In this updated form of Equation (10); **$C_{NW}$**, **$C_{N}$**, **$C_{NE}$**, **$C_{W}$**, **$C_{E}$**, **$C_{SW}$**, **$C_{S}$**, and **$C_{SE}$** are the state variables that can have the values either **1** or **0**. Therefore, the particular state variable will contribute in the XOR operation or state update process only if its respective bit will be equivalent to **1**, otherwise it will not take part in the state update process.

Thus, the structural combination of above direction variables based on the binary value of decimal number is employed to elect which and whom cells/cell will take part in the state update process. Hence different binary representation means different confined rule equation for the matrix update process of each succeeding matrix. The general formula of the confined rules is given below.

(11) $$CA_{CR}=\left\{\begin{array}{cc} {}[2\left(ICM_{\alpha }^{t}+ICM_{\beta }^{t}\right)]modF+1 & T=1\\ {\left(T-1\right)}_{\alpha }+{\left(T-1\right)}_{\beta }]modF+1 & T>1 \end{array}\right.$$

In this formula α = *count* $0^{s}\times 48$, β = *count* $1^{s}\times 49$, $ICM_{i}^{t}$ is the initial configuration matrix for the green channel that we got from the reserved hash (SHA-512) values for the green channel. *F* is the highest value of pixel that is 255. Whereas, *T* is the total number of blocks. For clear demonstration, let suppose we have the initial configuration matrix $C^{0}$ with a size 4×8 as given in Table 3.

From the ASCII table as the value of **1** is **49** and the value of **0** is **48**, so the decimal value for the confined rule structure of the **1^*st*^** sub-matrix can be found as follows.

$$CA_{CR}=\left[\left(12\times 48\right)+\left(20\times 49\right)\right]mod255+1$$

$$\Rightarrow CA_{CR}=26+1=27$$

Now converting this **27** into the binary form **[00011011]_2_**. As in this binary representation only **4** bits are **1**, So only these **4** cells will participate in the XOR operation of 1^*st*^ sub-matrix. To find those specific **four** cells we now put this binary value in Equation (10).

(12) $$\begin{array}{c} C_{M(i,j)}^{t+1}=\left(0\times C_{i-1,j+1}^{t}\right)⊕\left(0\times C_{i,j+1}^{t}\right)⊕\left(0\times C_{i+1,j+1}^{t}\right)⊕\left(1\times C_{i-1,j}^{t}\right)⊕\left(1\times C_{i+1,j}^{t}\right)⊕(0\\ \times C_{i-1,j-1})⊕\left(1\times C_{i,j-1}\right)⊕\left(1\times C_{i+1,j-1}\right) \end{array}$$

So Equation (12) will be simplified as follows.

(13) $$\begin{array}{c} C_{M(i,j)}^{t+1}=C_{i-1,j}^{t}⊕C_{i+1,j}^{t}⊕C_{i,j-1}⊕C_{i+1,j-1} \end{array}$$

This updated Equation (13) has four state variables that are $C_{W}$ = 1, $C_{E}$ = 1, $C_{S}$ = 1 and $C_{SE}$ = 1. So, only these four cells will contribute in the state update process of the 1^*st*^ sub-matrix. From Table 2 we can see $C_{i-1,j}^{t}$ is actually **$C_{W}$**, $C_{i+1,j}^{t}$ is actually **$C_{E}$**, $C_{i,j-1}^{t}$ is actually **$C_{S}$**, while $C_{i+1,j-1}^{t}$ is actually **$C_{SE}$**. Hence, by means of these confined rules structure, the processing power, computation time, efficiency and the security can be improved immensely.

The initial configuration matrix gets updated based on these confined rules mapping and then XORed with the particular plain image sub-matrix to acquire the updated matrix.

(14) $$C_{k(i,j)}^{t+1}=C_{M(i,j)}^{t+1}⊕C_{k(i,j)}^{t}$$

Here $C_{k(i,j)}^{t+1}$ is the updated matrix, $C_{M(i,j)}^{t+1}$ is the updated initial configuration based on confined rules, and $C_{k(i,j)}^{t}$ is the plain image matrix. In decryption process the reverse equation to get back the original plain sub-matrix will be as follows.

(15) $$C_{k(i,j)}^{t}=C_{M(i,j)}^{t+1}⊕C_{k(i,j)}^{t+1}$$

### 2.4. DNA Sequence

DNA molecule contain the genetic information that is used for the purpose of reproduction, functioning and growth of all living organism. Any DNA sequence comprised of four nucleic acid bases: Adenine (**A**), Cytosine (**C**), Guanine (**G**), and Thymine (**T**). These bases follow the principle of Watson–Crick, that is A and T are complementary, so are C and G same as in the binary system, **1** and **0** are complementary.

Likewise, the case of two-bits binary **11** and **00** are also complementary. Usually in DNA sequence every base is represented by the two-bits as 11, 10, 01 and 00 to represent these four bases T, G, C, and A respectively. There are basically **24** different kinds of DNA coding schemes. Nonetheless, out of those, only eight kinds fulfill the Watson–Crick complementary principle [51], which are given in Table 4. Please note that, like DNA encoding, the DNA decoding rules are just the inverse process of the DNA encoding rule.

**Figure 5:**
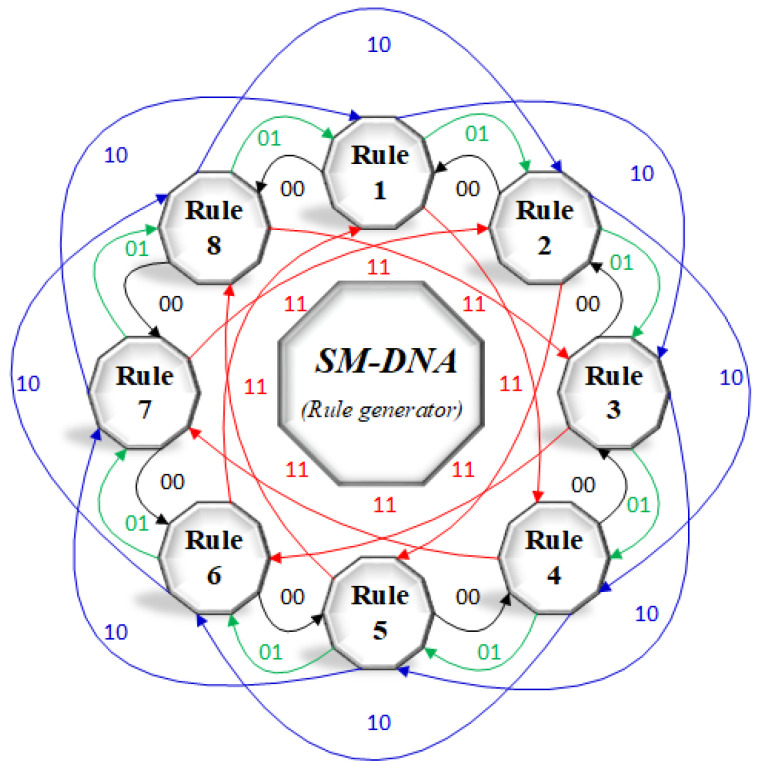
SM-DNA (Random rule generator).

In this paper, we propose a new bidirectional mathematical model of DNA rule generator based on two bits of input as shown in Figure 5. The working principle of this model is based on Automaton state machine, so it is called the **SM-DNA** rule generator. SM-DNA generates random DNA rules faster than the chaotic serious-based rules selection method.

The pixel value of image matrix was converted into binary, then divided into sub-matrices form. We used $i^{th}$ and $j^{th}$ bits as an input. SM-DNA select random DNA rule for each block matrix. In our case, the first and last bit of every sub-block matrix was used as an input. The proposed SM-DNA model has eight states and each state represents one rule. Each state has **2^2^** possible output states that take to next or previous state, so it means 8^4^ = **4096** different rules combination, while rule transition from one to another depends solely on the following **4** combinations, either **11**, **10**, **01** or **00**. For 00 it moves anti-clockwise direction and for 01, 10 and 11 it moves clockwise direction.

**Figure 6:**
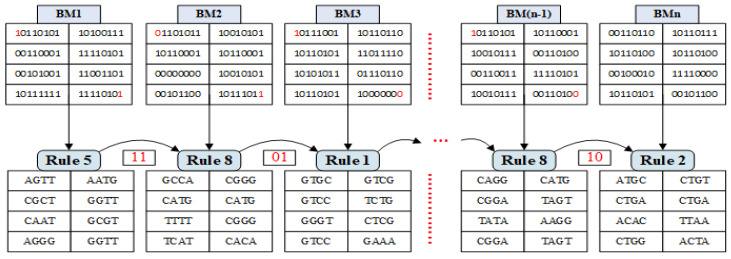
Binary to DNA Conversion.

For ease of implementation binary matrix of the image was converted into 4×8 sub-matrices. SM-DNA allocated random DNA conversion rule to each sub-matrix very efficiently and quickly. For more clear understanding, working principle illustration is given in Figure 6 with a total of *N* binary sub-block matrices termed as $B_{1},B_{2},B_{3},\ldots ,B_{\left(n-1\right)},B_{n}$. So the general formula of SM-DNA described as follows.

(16) $$SM:DNA=\left\{\begin{array}{cc} {}[\left(NP_{1}+NP_{2}\right)]modB+1 & N=1\\ {\left(N-1\right)}_{k^{th}}+{\left(N-1\right)}_{n^{th}}]modB+1 & N\neq 1 \end{array}\right.$$

where **k^*th*^** and **n^*th*^** are the two particular bits of own choice to serve as an input for the rule selection, that is $first^{bit}$ and $last^{bit}$ in our case. Suppose $BM_{1st}$ is our first block matrix and also suppose the **P_1_** and **P_2_** value came out **109** and **207**, respectively. So DNA starting rule for the 1st sub-block matrix can be gotten as follows.

$$SM:DNA_{1}=\left(1\times 109+1\times 207\right)mod8+1=5$$

So, **Rule 5** will be the starting rule that will be used for binary to DNA conversion of 1^*st*^ sub-block matrix.

$$BM_{1st}::\left[\begin{array}{cccc} 10 & 11 & 01 & 01\\ 00 & 11 & 00 & 01\\ 00 & 10 & 10 & 01\\ 10 & 11 & 11 & 11 \end{array}\right]\vec{Rule5}::\left[\begin{array}{cccc} A & G & T & T\\ C & G & C & T\\ C & A & A & T\\ A & G & G & G \end{array}\right]$$

For the 2^*nd*^ sub-block matrix $i^{th}=first^{bit}$ and $j^{th}=last^{bit}$ bit of 1^*st*^ block matrix will be used that are **1,1**. So, the **Rule 8** is the conversion rule for 2^*nd*^ sub-block matrix.

$$BM_{2nd}::\left[\begin{array}{cccc} 01 & 10 & 10 & 11\\ 10 & 11 & 00 & 01\\ 00 & 00 & 00 & 00\\ 00 & 10 & 11 & 00 \end{array}\right]\vec{Rule8}::\left[\begin{array}{cccc} G & C & C & A\\ C & A & T & G\\ T & T & T & T\\ T & C & A & T \end{array}\right]$$

As input bits of the 2^*nd*^ sub-block matrix are **0,0**, So it went to state **7**. Thus, **Rule 7** will be the conversion rule for the 3^*rd*^ sub-block matrix.

$$BM_{3rd}::\left[\begin{array}{cccc} 10 & 11 & 10 & 01\\ 10 & 11 & 00 & 01\\ 00 & 10 & 01 & 11\\ 10 & 00 & 10 & 00 \end{array}\right]\vec{Rule7}::\left[\begin{array}{cccc} G & A & G & C\\ G & A & T & C\\ T & G & C & A\\ G & T & G & T \end{array}\right]$$

Similarly, all the sub-block matrices were converted into DNA sequences based on rules generated by SM-DNA as shown in Figure 6. Remember that for the $N^{th}$ sub-block matrix the ${\left(N-1\right)}^{th}$ sub-block matrix will decide the rule. From security prospective as rule selection based on two bits inputs so it is very difficult to predict that out of 32-bits, which two-bits are working as an input. Thus its difficult to guess the rule selection without getting adequate information about the working principle of SM-DNA.

## 3. Proposed Encryption Method

**Figure 7:**
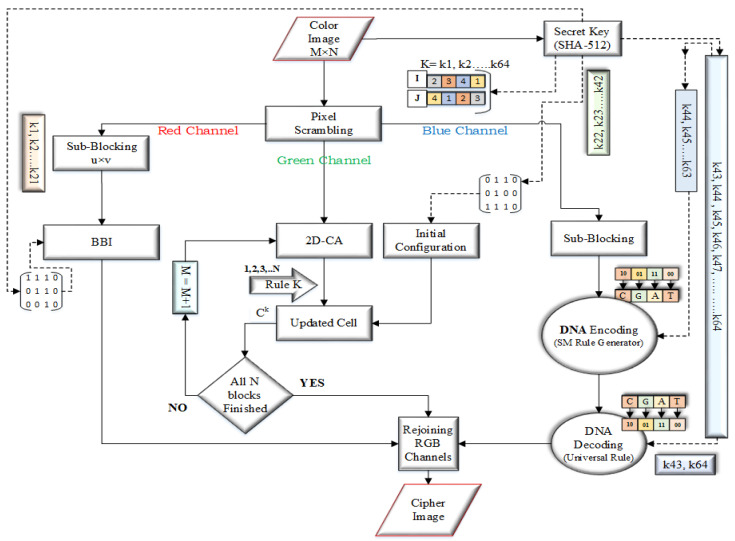
General Block Diagram

The general block diagram of the proposed image encryption method is shown in Figure 7. The proposed encryption method deal with the binary bit-planes of each channel. The detailed encryption steps are given below.

**Step 1**: Get the double hash-value of the image by using SHA-512. Hexadecimal forms of the key string will be gotten. Split the key string into three parts i.e., $K_{1}=21$, $K_{2}=21$ and $K_{3}=22$ hexadecimal pairs to use in the formulas and initial configuration matrices of red, green and blue channels as follows.

$$K_{red}=k_{1},k_{2},k_{3},k_{4},\ldots ,k_{20},k_{21}$$

$$K_{green}=k_{22},k_{23},k_{24},k_{25},\ldots ,k_{41},k_{42}$$

$$K_{blue}=k_{43},k_{44},k_{45},k_{46},\ldots ,k_{63},k_{64}$$

**Step 2**: Take the color image (M,N,3), where *M* and *N* denote the rows and columns of the image, respectively. Scramble as described in the scrambling portion, while the hexadecimal form of a key was used as a seed to generate array sequence by LSS−PRNG. For the two rounds of sequences, the seed values are set through the following equations.

(17) $$\begin{array}{c} x_{0}=\frac{\left(k_{1}k_{2}⊕k_{3}k_{4}\right)+\ldots +\left(k_{13}k_{14}⊕k_{15}k_{16}\right)}{256} \end{array}$$

(18) $$\begin{array}{c} y_{0}=\frac{\left(k_{17}k_{18}⊕k_{19}k_{20}\right)+\ldots +\left(k_{29}k_{30}⊕k_{31}k_{32}\right)}{256} \end{array}$$

(19) $$\begin{array}{c} z_{0}=\frac{\left(k_{33}k_{34}⊕k_{35}k_{36}\right)+\ldots +\left(k_{45}k_{46}⊕k_{47}k_{48}\right)}{256} \end{array}$$

The integer values for two round from the $n^{bits}$ of the stream gotten by the following formula.

(20) $$integer=\sum_{i=1}^{n}b_{i}\times 2^{i-1}$$

For the two rounds of array sequence the initial state can be set by the following way.

(21) $$\begin{array}{c} X_{0}^{i}=\left[integer\times y_{0}\times \left(x_{0}+z_{0}\right)\right]mod1\\ r^{i}=\left[integer\times x_{0}\times \left(y_{0}+z_{0}\right)\right]mod4 \end{array}$$

where, i=(1,2) termed as rounds and it will generate two initial values $X_{0}^{1}$,$r^{1}$ and $X_{0}^{2}$,$r^{2}$ respectively.

**Step 3**: Split the scrambled color image $T_{\left(M,N,3\right)}^{Scr.}$ into respective three channels R, G and B channels and we can get the three components, $T_{R}^{Scr.}$, $T_{G}^{Scr.}$ and $T_{B}^{Scr.}$ as given below.

(22) $$T_{R}^{Scr.}=T_{r1},T_{r2},T_{r3},\ldots ,T_{rMN}$$

(23) $$T_{G}^{Scr.}=T_{g1},T_{g2},T_{g3},\ldots ,T_{gMN}$$

(24) $$T_{B}^{Scr.}=T_{b1},T_{b2},T_{b3},\ldots ,T_{bMN}$$

Here $T_{ri}$, $T_{gi}$ and $T_{bi}$ are the $i^{th}$ pixel values of the red, green and blue channels respectively, whereas $T_{ri};T_{gi};T_{bi}\in \left[0,255\right]$. Transformed the pixel values of each channel into the binary windows as given below.

(25) $$T_{R(M,N)}=R_{w1},R_{w2},R_{w3},\ldots ,R_{w8}$$

(26) $$T_{G(M,N)}=G_{w1},G_{w2},G_{w3},\ldots ,G_{w8}$$

(27) $$T_{B(M,N)}=B_{w1},B_{w2},B_{w3},\ldots ,B_{w8}$$

Now split the window matrix into m×n size sub-blocks, where u×m = *M* and v×n = *N*.

**Step 4**: Take the $1^{st}$ block of $T_{R(M,N)}$ with a size 8×8 and split it into two equal columns block e.g., 8×4 and 8×4; termed as LCB and RCB. By using formula get the starting bit for the 1^*st*^ block-matrix and apply block bit inversion as described in Section 2.2.

**Step 5**: Take the green channel $T_{G(M,N)}$ and apply the Cellular Automata as described in Section 2.3.

**Step 6**: Take the blue channel $T_{B(M,N)}$ of size H×W divide it into the u×v size sub-blocks matrices as given below. While u×H=M and v×W=N, and apply DNA conversion as described in Section 2.4.

(28) $$T_{B(M,N)}^{bi}=BM_{1}^{bi},BM_{2}^{bi},BM_{3}^{bi},\ldots ,BM_{{\left(n-1\right)}^{th}}^{bi},BM_{n^{th}}^{bi}$$

With SM-DNA convert every binary block matrix into DNA matrix based on allocated rule of SM-DNA as follows.

$$BM_{1}^{bi}⇌BM_{1}^{DNA},BM_{2}^{bi}⇌BM_{2}^{DNA},BM_{3}^{bi}⇌BM_{3}^{DNA}$$

$$,\ldots ,BM_{\left(n-1\right)}^{bi}⇌BM_{\left(n-1\right)}^{DNA},BM_{n^{th}}^{bi}⇌BM_{n^{th}}^{DNA}$$

Join all these DNA sub-block matrices into a single matrix as described below.

(29) $$BM_{1}^{DNA},BM_{2}^{DNA},BM_{3}^{DNA},\ldots ,BM_{n^{th}}^{DNA}=T_{B(M,N)}^{DNA}$$

Get the universal DNA→binary rule through following formula.

(30) $$DNA_{UR}=\left[P_{1}+P_{2}\right]mod8+1$$

Convert the whole DNA sequence matrix back into binary form through this universal rule.

**Step 7**: Add the random matrix of the scrambling part to the pixel values of each channel as described below.

(31) $$\begin{array}{c} Cipher_{RGB}↔\left\{\begin{array}{c} C_{\left(i,j\right)}^{red}=\left[E_{\left(i,j\right)}^{red}+R_{\left(i,j\right)}\right]mod256\\ C_{\left(i,j\right)}^{green}=\left[E_{\left(i,j\right)}^{green}+R_{\left(i,j\right)}\right]mod256\\ C_{\left(i,j\right)}^{blue}=\left[E_{\left(i,j\right)}^{blue}+R_{\left(i,j\right)}\right]mod256 \end{array}\right. \end{array}$$

where $C_{(}i,j)$ denotes cipher value of particular pixel, $E_{(}i,j)$ denotes encrypted value of the pixel, $R_{(}i,i)$ denotes the Random matrix, i=1,2,…M, and j=1,2,…,N.

**Step 8**: Rejoin all the channels to get the cipher image.

(32) $$Cipher_{\left(M,N,3\right)}=C_{\left(i,j\right)}^{red};C_{\left(i,j\right)}^{green};C_{\left(i,j\right)}^{blue}$$

## 4. Experimental Results and Discussion

This section included the performance and simulation results along with the comparison of results with earlier proposed image encryption schemes. The experimental results were manipulated in **Python 3.6.5** (Jupyter Notebook Environment) installed over a personal Laptop, CPU Intel **Core I5** with **4GB** memory and operating system **Window 10**.

**Figure 8:**
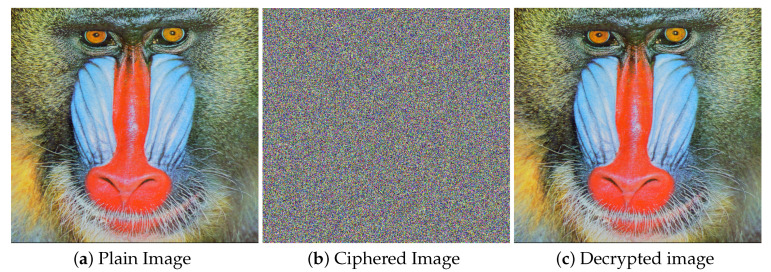
Encryption and decryption results. (**a**) The plain image of baboon; (**b**) The cipher image of baboon; (**c**) The decrypted image of baboon.

**Figure 9:**
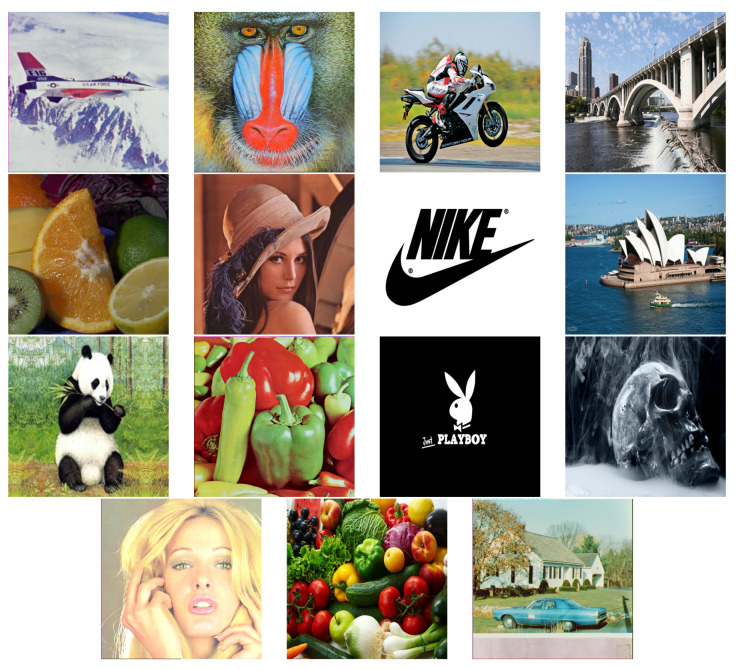
All the test Images.

The test image baboon, its cipher image and the decrypyted image are shown in Figure 8. The experimental parameters are given in Table 5. Whereas all the test images that we used for experiments are shown at the end of paper in Figure 9.

## 5. Security Analysis and Test

### 5.1. Security Keyspace

The keyspace represents the entire number of likely combinations of the security key. The most common attack is the Brute-force attack in which an attacker endeavors to predict the accurate security key by overly searching the keyspace of the encryption algorithm. Thus, in order to withstand against the Brute-force attack, an adequately huge keyspace is one of the main factor that can guarantee more security [52].

For resisting the best attack, secure hash algorithm (SHA-512) uses a keyspace of **2^256^**. The comparison of keyspace and approach along with testing parameters of the proposed algorithm is given in Table 6.

Moreover, the SM-DNA possible rules combination **8^4^** can also be taken as keyspace. Except for the fact that the hash keys for generating initial configuration matrices for each channel and in their particular rule generating formulas are **2^22^**, **2^22^**, **2^21^**, and **2^5^** respectively. So, the overall keyspace of the proposed algorithm is $2^{256}\times 2^{21}\times 2^{22}\times 2^{22}\times 2^{5}\times 8^{4}$ which is very large as compared to **2^128^**. To calculate the computational load, let’s the fastest computer computes $2^{80}$ computations in **1** second and $2^{80}\times 365$(days)×24(h)×60(min)×60(s) [57]. So, to compute $8^{4}\times 2^{>310}$ computations, a total of following years required.

$$\frac{8^{4}\times 2^{>310}}{2^{80}\times 365\times 24\times 60\times 60}\Rightarrow \approx 2.68\times 10^{65}years$$

This huge computation load is sufficient enough to break the crypto-system. The computational load also proved that the **3C3R** can effectively withstand against the brute-force attacks.

### 5.2. Histogram Analysis

**Figure 10:**
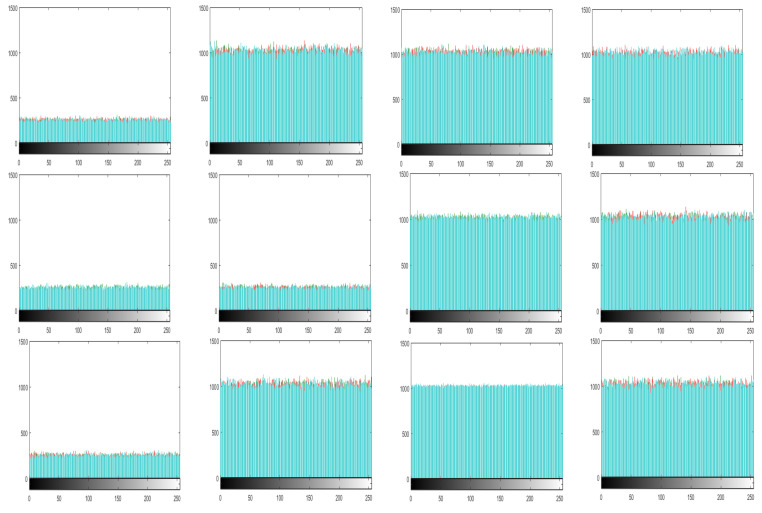

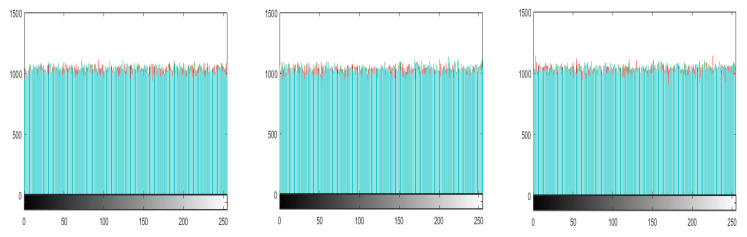
Histogram of ciphered images of the above all the test Images in Sequence.

**Figure 11:**
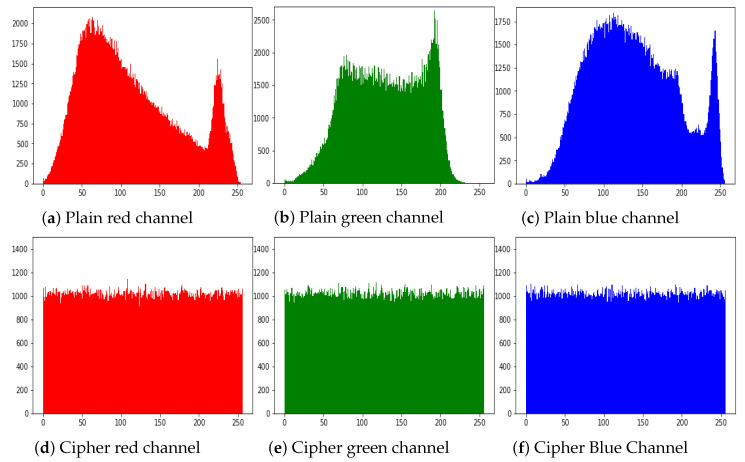
Histograms of RGB channels of Baboon. (**a**) Plain red channel; (**b**) Plain green channel; (**c**) Plain blue channel; (**d**) Ciphered red channel; (**e**) Ciphered green channel; (**f**) Ciphered blue channel.

The histogram of an image demonstrate the distribution of the pixel values. An intruders usually recover the meaningful information from the fluctuating histogram of the encrypted image. So, in order to prevent an intruder from recovering such information, it is important that the histogram of the cipher-image should have no statistical resemblance to the plain image and also should have uniform distribution. The histograms of the ciphered images of all the test images are shown in Figure 10. While Figure 11 shows the histogram of RGB channels of the plain image and its corresponding cipher image. The histogram of the each RGB channel of the cipher image is almost uniform. Moreover, by computing the variance of histogram, we evaluated the uniformity of our ciphered images. The lesser the variance means the higher is the uniformity of the encrypted images [58,59,60,61].

(33) $$var\left(I\right)=\frac{1}{k^{2}}\sum_{i=1}^{k}\sum_{j=1}^{k}\frac{1}{2}{\left(I_{i}-I_{j}\right)}^{2}$$

Table 7 listed the histogram variances of the plain images (R,G,B) and encrypted images along with a comparison with the other methods. From the table, we can see that in most of the test images the histogram variance of the proposed algorithm is less as than from Ref. [62] and Ref. [49]. This proved that the **3C3R** has better security in comparison to those algorithms.

### 5.3. Pixel Correlation Analysis

Pixel correlation analysis is another test used to find the relationship of neighboring pixels in the plain image and the ciphered image. A good encryption algorithm aims to minimize the relationship among the neighboring pixels with regards to prevent the leakage of actual information.

The correlation coefficient ***C_r(x,y)_*** between the two neighboring pixels can be calculated by the following formulas.

(34) $$E\left(w\right)=\frac{1}{P}\sum_{i=1}^{P}w_{i}$$

(35) $$D\left(w\right)=\frac{1}{P}\sum_{i=1}^{P}{\left(w_{i}-E\left(w\right)\right)}^{2}$$

(36) $$C_{ovariance}\left(w,z\right)=\frac{1}{P}\sum_{i=1}^{P}\left(w_{i}-E\left(w\right)\right)\left(z_{i}-E\left(z\right)\right)$$

(37) $$C_{r(w,z)}=\frac{C_{ovariance}\left(w,z\right)}{\sqrt{D(w)\times D(z)}}$$

**Figure 12:**
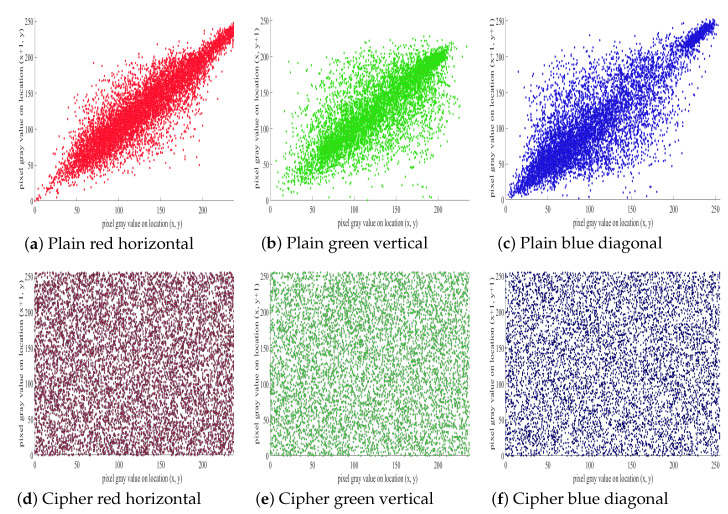
Correlation of RGB Channels of baboon (**a**) Plain red in horizontal; (**b**) Plain green in vertical; (**c**) Plain blue in diagonal; (**d**) Ciphered red in horizontal; (**e**) Ciphered green in vertical; (**f**) Ciphered blue in diagonal.

In above equations (***w, z***) is the gray values of neighboring pixels, ***C_ovariance_(w,z)*** is the covariance, ***P*** is the total number of pixels selected from the image, while ***E(w)*** is the mean and ***D(w)*** is the variance. Figure 12 displays the pixels of the plain image and ciphered image of the proposed algorithm in horizontal (H), vertical (V) and diagonal (D) distribution. In Table 8 and Table 9 the pixel correlation comparison was done with some previous algorithms. Table 8 shows a comparison of **8K** pairs of neighboring pixels that are randomly selected from the plain image and the ciphered image in the H, V, and D directions to perform pixel correlation analysis. Whereas, Table 9 shows a correlation comparison of **1K** random pixels of the Lena image. Whereas, Table 10 listed the pixel correlation values of the different test images.

We casually selected the pixel pairs in the horizontal, vertical and diagonal axes, respectively. From Table 9 it is obvious that in term of overall correlation, proposed **3C3R** and Ref. [63] are performing well, while in term of diagonal values our algorithm, Refs. [63,64,65] are giving satisfactory values. While the overall and in terms of **UACI** and **NPCR**, the proposed **3C3R** outperformed.

Similarly, for **8K** random pixels the Refs. [55,58,66,68,70] are giving good results but the proposed **3C3R** is also performing well in regards to vertical and diagonal direction.

### 5.4. Key Sensitivity Analysis

Because of the enhanced computational power, the current era’s encryption algorithms should not have key length less than 100 bits or ($2^{100}$). Such key length can withstand against the exhaustive key search attack (brute force attack). The keyspace of the proposed encryption method is $2^{\left(>312\right)}$, which has high ability to resist the brute-force attack. Except for the fact that the key should also be extremely sensitive to the bit change. If the secret key will not be subtle enough, then a slight change in the actual secret keys can also properly recover the original image. Also, the secret key may perverted and as a result the actual keyspace may less than the theoretical one [60,61,78].

So, to check the sensitivity of the proposed algorithm towards the key, experiment has performed by changing one bit in the key with respect to the actual key as shown below.

**Key_o_** = “40 CD 74 4F 66 82 BD 0A CF 73 57 9A 5D C3 53 DB 3A 29 5D 3A 2D 87 03 56 6C 8A CF 9B E8 AA 68 8E 87 62 1E 8F 5F 3D 07 37 63 C4 6E 93 FF 7B 1A 2B 04 76 C3 BB 84 08 F2 A2 E8 AF AB 48 08 7B B9 C4”.

**Key_1_** = “40 CD 74 4F 66 82 BD 0A CF 73 57 9A 5D C3 53 DB 3A 29 5D 3A 2D 87 03 56 6C 8A CF 9B E8 AA 68 8E 87 62 1E 8F 5F 3D 07 37 63 C4 6E 93 FF 7B 1A 2B 04 76 C3 BB 84 08 F2 A2 E8 AF AB 48 08 7B B9 C5”.

**Key_2_** = “50 CD 74 4F 66 82 BD 0A CF 73 57 9A 5D C3 53 DB 3A 29 5D 3A 2D 87 03 56 6C 8A CF 9B E8 AA 68 8E 87 62 1E 8F 5F 3D 07 37 63 C4 6E 93 FF 7B 1A 2B 04 76 C3 BB 84 08 F2 A2 E8 AF AB 48 08 7B B9 C4”.

**Figure 13:**
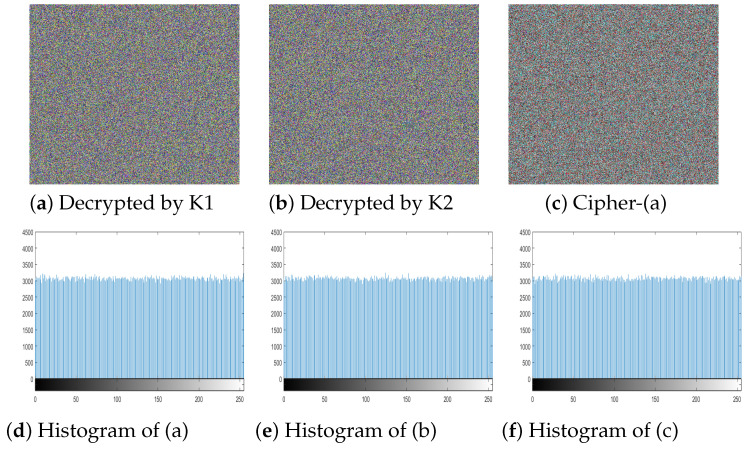
Key sensitivity test. (**a**) Ciphered image decrypted by K1; (**b**) Ciphered image decrypted by K2; (**c**) Subtracted image of cipher-(a); (**d**) Histogram of (a); Histogram of (b); Histogram of (c).

**Key_o_** is the actual key while **Key_1_**, **Key_2_** are the one-bit changed keys from LSB (Right) and MSB (left) bit respectively. Figure 13a,b show the decrypted image by **Key_1_** and **Key_2_** respectively. Both **Key_1_** and **Key_2_** are one bit different from the actual key but the decrypted image is still like noise giving no useful information. The Figure 13c is the subtracted image of the actual cipher and the decrypted image of **Key_1_**. The histogram of particular three resultant images is given in Figure 13d–f respectively. The subsequent uniform histograms proved that the proposed **3C3R** is very sensitive to key change even one-bit change leads towards totally different cipher image.

### 5.5. Differential Attack

Normally, hackers; to extract useful information create little modification in the plain image and then by using the encryption methodology they encrypt the identical images afore and afterwards these slight changes. Through this method, they attempt to find out the association among the plain images and the cipher images. Thus, we employed the number of pixel change rate **(NPCR)** and unified average changing intensity **(UACI)** to measure the robustness of the proposed algorithm against such attacks. The **NPCR** and **UACI** can be calculated by the following way.

(38) $$NPCR_{U_{1},U_{2}}=\left[\frac{\sum_{r,c}I\left(r,c\right)}{H\times M}\right]\times 100$$

(39) $$UACI_{U_{1},U_{2}}=\frac{1}{H\times M}\left[\sum_{r,c}\frac{|U_{1}\left(r,c\right)-U_{2}\left(r,c\right)|}{2^{8}-1}\right]\times 100$$

Here ***U*_1_**, ***U*_2_** are two different ciphered images before and after one pixel of the plain image is changed, while H×M is the height and width of the test image. Whereas, ***I(r, c)*** can be defined as

(40) $$I\left(r,c\right)=\left\{\begin{array}{cc} 0 & U_{1}\left(r,c\right)\neq U_{2}\left(r,c\right)\\ 1 & otherwise \end{array}\right.$$

In the above equation, ***I*** depict the difference between ***U*_1_** and ***U*_2_**.

Table 11 listed the NPCR and the UACI values of different test images along the comparison with some earlier algorithms. From the table values, we can see the test results of the **Lena** and **pepper** images of our **3C3R** giving **NPCR** ≥ **99.97** and **UACI** ≥ **33.46**. NPCR is high from Refs. [28,58,59,79,80], while it is comparable to [81]. Similarly, UACI is also comparable with the Refs. [59,79,81]. Thus the proposed **3C3R** has satisfactory security values.

### 5.6. Known and Chosen Plain Text Analysis

Most commonly, four kind of cryptanalsis attacks can be performed to crack the image encryption algorithms that are chosen-cipher-text attack, chosen-plaintext attack, cipher-text only attack, and known-plaintext attack [82]. Some famous image encryption algorithms given in Table 1 have already been broken with these attacks.

The cryptanalysis model, where the attackers choose plaintext to obtain the corresponding cipher-text is called chosen-plain text attack. By examining the plaintext and the corresponding cipher-text, they try to presume some hidden useful information. Finally, by using that information they try to recover the original images [85,86].

The chosen-plaintext attack is the most powerful, and if the encryption may resist this attack, it has adequate security level to withstand the other three attacks. The proposed algorithm **3C3R** has satisfactory security level against known and chosen plaintext attacks.

**Figure 14:**
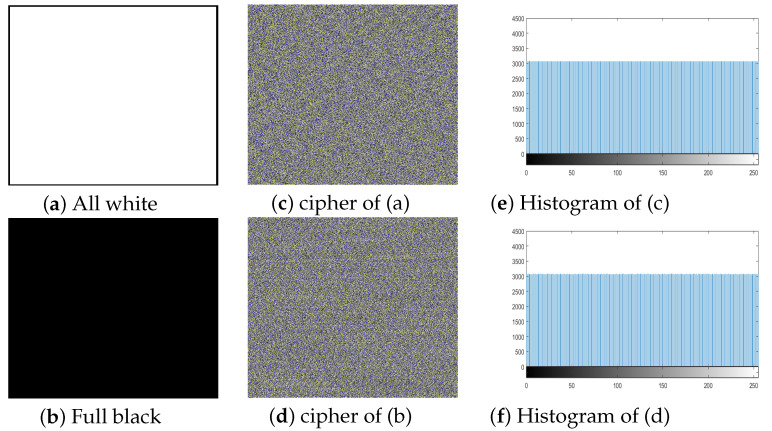
Encryption result of all white and full black images. (**a**) All white image; (**b**) Full black image; (**c**) Cipher image of (a); (**d**) Cipher image of (b); (**e**) Histogram of (c); (**f**) Histogram of (d).

We can see the cumulative entropy_(R,G,B)_ value is **8.00**. In Table 15 comparison of the entropy values was also given, the value is higher from Refs. [82,87,88,89]. The proposed algorithms give the ideal all channels entropy values for all white, full black image and is given in Table 16. The proof of ideal entropy value is visible in the histogram of the ciphered image generated by our **3C3R** of all white and full black, and was given in Figure 14. The histogram of all white, full black and Playboy image is almost entirely flat. Furthermore, we created two special color images SP_image1_ and SP_image2_ respectively of size P×Q×3. SP_image1_ is the color image with all pixels’ values **0** except one pixel located at (252, 252) in R channel is 1. Similarly SP_image2_ is the color image with all pixels’ values **1** except one pixel located at (252, 252) in R channel is 0. We made plaintext sensitivity analysis and the results are given in Table 12. The average values of NPCR and UACI are closed to the theoretical value. Thus on the bases of this test we can say that **3C3R** is robust against such attacks and can keep the image more secure.

### 5.7. Robustness against Occlusion Attack

In image processing, PSNR and MSE are the most widely used parameters to test the encryption quality. The PSNR and MSE values can be calculated as follows.

(41) $$PSNR=10\times log_{10}\left(\frac{255\times 255\times 3}{MSE_{R}+MSE_{G}+MSE_{B}}\right)\left(dB\right)$$

(42) $$MSE=\frac{1}{MN}\sum_{r}^{M}\sum_{c}^{N}{∥I_{p}\left(r,c\right)-I_{c}\left(r,c\right)∥}^{2}$$

where $MSE_{R,G,B}$ is the mean square error of red, blue and green channel, between the cipher image $I_{c}\left(r,c\right)$ and original image $I_{p}\left(r,c\right)$, while *M* and *N* is the height and width of the image respectively.

**Figure 15:**
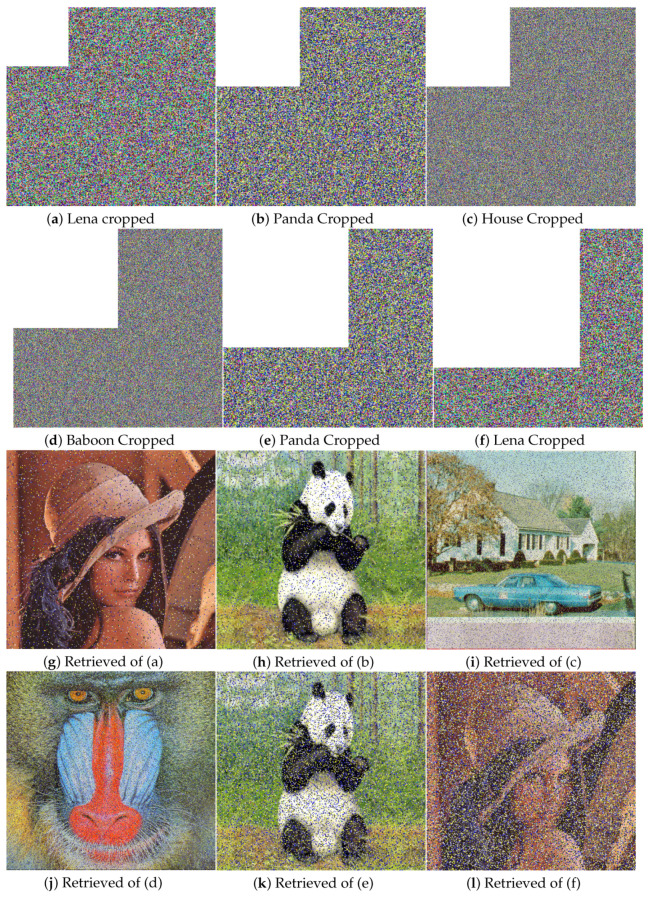
Occlusion attack test. (**a**) Cropped image of Lena; (**b**) Cropped image of Panda; (**c**) Cropped image of House; (**d**) Cropped image of baboon; (**e**) Cropped image of Panda; **(f**) Cropped image of Lena; (**g**) Retrieved image of (a); (**h**) Retrieved image of (b); (**i**) Retrieved image of (c); (**j**) Retrieved image of (d); (**k**) Retrieved image of (e); (**l**) Retrieved image of (f).

Another way is quality checking at the receiver side, such as after passing through a noisy medium cipher, an image may get blurred or lose some data. Therefore, a trustworthy encryption scheme should be able to recuperate the original image without losing too much substantial information. Figure 15a–f shows the different test images with different cropped portion. While Figure 15g–l are the retrieved images, we can see the retrieved images are easily recognizable and carry good information even after clipping 1/2. While the less clipping gave a much better result. Table 13 listed the PSNR and MSE comparison with [29] of test image Lena with different proportion of cropping. The values show that our algorithm performs better when increased the clipping portion while values are comparable for the less clipping.

Measuring the difference between the cipher image and original image is another way to evaluate the quality of the color images. So, for this purpose the PSNR can be viewed as a security evaluation parameter. The encryption effect is consider better if the value of PSNR is lower. Table 14 listed the PSNR value between plain↦decrypted and plain↦ciphered images. The comparison was also made with some well-known algorithms and values are listed in the particular table. We can see from the table that the PSNR value for the different test images is PSNR≤**8.10** that is lower than Refs. [15,23,46,90,91] except the test image baboon in which Ref. [15] gave the lowest PSNR value between plain image and ciphered image. Thus, based on the PSNR value test, we can say that our **3C3R** performs very well and can guaranty more security in comparison to other algorithms.

### 5.8. Local and Shannon Information Entropy

The information entropy (IE) defines the degree of disorder or chaos in an encryption system through the gray value probability. IE for the image can be defined as follow:

Let an information source be a τ, then IE can be computed as follows.

(43) $$H\left(\tau \right)=\sum_{i=0}^{2^{n}-1}\rho \left(\tau_{i}\right)log_{10}\frac{1}{\rho (\tau_{i})}$$

Here $\rho (\tau_{i})$ depicts the probability of the symbol $\tau_{i}$. The ideal IE value for the image with gray intensity level of 2^8^ is **8** [93]. So, it means the closer the IE value, the more is the randomness of an image, and as a result less information will be revealed by the particular encryption scheme. Table 15 and Table 16 enlisted the entropy value of some famous test images and their comparison with some earlier encryption algorithms. The table values are the evidence of **IE** ≥ **7.996**, that is close enough to the ideal value **8.0**. While for Playboy, full white and Full black **3C3R** achieved the ideal value **IE = 8.00**. IE values of all the test images are higher than Refs. [82,87,88,89].

In [94] a new image uncertainty test introduced by means of Shannon entropy over the native image-blocks. The **(k, T_P_)** Shannon entropy measure concerning local image blocks can be calculated by the following method:

**Step 1:** Select the non-overlapping image blocks randomly i.e., $B_{1},B_{2},B_{3},\ldots ,B_{k}$ with T_P_ Pixels within the ciphered or test image *I* with intensity scales *L*.

**Step 2:** Compute Shannon entropy for all i∈(1,2,3,…,k) by using Equation (44).

**Step 3:** Calculate the Shannon entropy sample mean over these **k** image blocks $B_{1},B_{2},B_{3},\ldots ,B_{k}$ by the following equation.

(44) $${\bar{H}}_{\left(k,T_{P}\right)}\left(B\right)=\sum_{i=1}^{k}\frac{H(B_{i})}{k}$$

The local Shannon entropy value was calculated for the ciphered images. Firstly, non-overlapping image blocks with k=32 and **T_P_** = **1936** pixels are randomly selected from the ciphered images. As the experiential value of local Shannon entropy must fall within the confidence interval i.e., **[7.9019, 7.9030]**, concerning the α-level sureness equal to the 0.05. Table 16 listed the Shannon entropy and global entropy values and also the comparison of Shannon entropy with Ref. [95]. The Shannon entropy value of the ciphered image of the proposed **3C3R** fully falls within the desired range. Thus, based on the ideal **IE** values, we can say the ciphered image generated by the proposed **3C3R** carries more haphazardness and as a result, assures more security.

### 5.9. Gray Value Degree (GVD) Analysis

The gray difference degree or GVD is another statistical test of haphazardness that can be found by comparing the plain image and the ciphered image. The ideal value is **1**, so closer the value the better is the security. GVD can be computed by the following Equation;

(45) $$G_{D}\left(r,c\right)=\sum \left[G\left(r,c\right)-G\left(r^{\prime },c^{\prime }\right)\right]$$

whereas G(r,c) symbolizes the gray score at position (r,c) and $\left(r^{\prime },c^{\prime }\right)$ is as given below.

(46) $$\left(r^{\prime },c^{\prime }\right)=\left\{\begin{array}{c} \left(r-1,c\right)\\ \left(r+1,c\right)\\ \left(r,c-1\right)\\ \left(r,c+1\right) \end{array}\right.$$

The average neighborhood gray difference of the whole image can be computed as follows.

(47) $$AV_{erage}\left[G_{D}\left(r,c\right)\right]=\frac{\sum_{r=2}^{M-1}\sum_{c=2}^{N-1}G_{D}\left(r,c\right))}{\left(M-2)(N-2\right)}$$

(48) $$AV_{erage}GVD=\frac{AV^{\prime }\left[G_{D}\left(r,c\right)\right]-AV\left[G_{D}\left(r,c\right)\right]}{AV^{\prime }\left[G_{D}\left(r,c\right)\right]+AV\left[G_{D}\left(r,c\right)\right]}$$

In these equations, $AN{}^{\prime }$ and AN, denotes the average neighborhood gray value; but the former represents after encrypting and the later represents before encryption. The final GVD value termed as gray value degree and it will be **1** if the two images are completely different or else it will be **0**, if the two images are same. The GVD score of the plain and encrypted images of **USC-SIPI** database are shown in Table 17. The GVD score for most of the test images is GVD ≥ **0.907** for each R, G and B channel which shows that the plain and encrypted images are entirely different. The listed results also show that **3C3R** ensures more security for images as compared to Refs. [29,69,97] except for the image **4.1.04** in which Ref. [29] has the higher value as compared to **3C3R**.

### 5.10. Performance Comparison

Performance and encryption time are an important characteristic of any image encryption algorithm. Like in the Chaos-based encryption schemes, number of permutation and diffusion rounds have a direct impact over the encryption time. Similarly for color image rule processing scheme over the RGB channels has a direct relation with the encryption time. Because of the parallel processing characteristics of cellular automata and DNA sequence the proposed **3C3R** takes less encryption time as compared to chaos-based schemes. Table 18 enlisted the time comparison of our **3C3R** with the Refs. [79,81,98,99] that possess a satisfactory security level. The least encryption and decryption time was taken by the proposed **3C3R** shows that our algorithm gives satisfactory results with minimum times. Except that Table 19 listed the comparison with the recently introduced algorithms. The table values in bold fonts clearly concludes that our **3C3R** overall performing much better than Refs. [56,62,76,91]. Hence, our **3C3R** assures better image security.

## 6. Conclusions and Future Work

To conquer the issue of low sensitivity to the secret key or low security against known plain or cipher-text attacks, this paper introduced a **3C3R** robust image encryption algorithm with adequate security level against well known attacks. Fully uniform histogram and ideal cumulative entropy **8.00** for some images are the proof of robustness and better security of an image by **3C3R**. Unlike most encryption methodologies in which the same encryption method or rules structure followed for all channels, this paper introduced a novel encryption method comprising different encryption strategy for each channels. Block bit inversion (binary) for the red channel, **VN** and **RVN** (cellular) structure-based pixel alteration for the green channel, and state machine-based **SM-DNA** rule allocation for the blue channel. Experimental results proved that our **3C3R** algorithm is highly subtle to the secret key along with better security. The proposed **3C3R** can keep different types of images safe and secure from attackers. **3C3R** outperforms state of the art algorithms in terms of encryption performance and image security. The experimental results also proved that the **3C3R** is robust against well-known attacks such as brute-force attacks, occlusion attacks, chosen/known plaintext attacks, and differential attacks. Therefore, we can say that **3C3R** is the algorithm of the current era requirement and hence it has potential applications in multimedia communication.

## Figures and Tables

**Table 1:** Algorithms that successfully get cryptanalyzed by well-know attack approaches.

Proposed by	Cryptanalysis by	Attack Approach
Zhang et al. (2013) [30]	Hoang et al. (2018) [31]	Chosen cipher-text
Zhou et al. (2015) [32]	Chen et al. (2017) [33]	Differential attack
Zhang et al. (2016) [34]	Wu et al. (2018) [35]	Chosen plaintext
Huang et al. (2012) [36]	Wang et al. (2014) [37]	Chosen plaintext
Chen et al. (2015) [38], Gao et al. (2008) [39]	Hu et al. (2017) [40], Rhouma et al. (2008) [41]	Chosen plaintext and cipher-text
Liu et al. (2016) [42], Tong et al. (2008) [43]	Zhang et al. (2017) [44], Li et al. (2009) [45]	Chosen plaintext
Zhu (2012) [46], Pak et al. (2017) [47]	Li et al. (2013) [48], Wang et al. (2018) [49]	Chosen plaintext

**Table 2:** VN + RVN Mapped Cell.

$C_{NW}$	$C_{N}$	$C_{NE}$
$C_{W}$	$C_{0}$	$C_{E}$
$C_{SW}$	$C_{S}$	$C_{SE}$

**Table 3:** Initial Configuration.

1	1	1	0	0	1	0	1
1	1	0	1	1	0	1	1
0	0	1	0	0	1	1	0
1	1	0	1	1	0	1	1

**Table 4:** DNA Rules.

**Rule 1**	**Rule 2**	**Rule 3**	**Rule 4**
00 ↦ **A**	00 ↦ **A**	01 ↦ **A**	01 ↦ **A**
11 ↦ **T**	11 ↦ **T**	10 ↦ **T**	10 ↦ **T**
01 ↦ **C**	10 ↦ **C**	00 ↦ **C**	11 ↦ **C**
10 ↦ **G**	01↦ **G**	11↦ **G**	00 ↦ **G**
**Rule 5**	**Rule 6**	**Rule 7**	**Rule 8**
10 ↦ **A**	10 ↦ **A**	11 ↦ **A**	11 ↦ **A**
01 ↦ **T**	01 ↦ **T**	00 ↦ **T**	00 ↦ **T**
00 ↦ **C**	11 ↦ **C**	01 ↦ **C**	10 ↦ **C**
11 ↦ **G**	00 ↦ **G**	10 ↦ **G**	01 ↦ **G**

**Table 5:** Experiment Parameters.

Terms	System Parameters/Values
512-bit	40CD744F6682BD0ACF73579A5DC353DB
Hexadecimal	3A295D3A2D8703566C8ACF9BE8AA688E
key	87621E8F5F3D073763C46E93FF7B1A2B
	0476C3BB8408F2A2E8AFAB48087BB9C4
Seed	int(K[0:2],16)=207
P1	int(K[45:48],16)=3086
P2	int(K[60:63],16)=1030
$P1_{universal}$	int(K[43],16)=12
$P2_{universal}$	int(K[64],16)=2

**Table 6:** Keyspace and Comparison with earlier.

Algorithm	Rule/Map	Keyspace	Operation	Image Type	Testing Parameters
Enayatifar^2017^ [53]	LM	120 bit	DNA XOR	Gray Scale	NPCR, UACI, Entropy, CC, Key-space, Histogram, Time parameter
Kumar^2016^ [54]	ILM	128 bit	CA	Color	NPCR, UACI, Entropy, CC, Key-space,
					Histogram, Noise test, Crop test
Guesmi^2016^ [55]	Lorenz	SHA-256	DNA XOR	Color	NPCR, UACI, Entropy, CC,
	system				Key-space, Histogram
S.Suri^2018^ [56]	ILM	SHA-256	DNA XOR	Color	NPCR, UACI, Entropy, CC,
			DNA Addition	Binary	Key-space, Histogram, Contrast
**Our Proposed 3C3R**			DNA,		NPCR, UACI, Entropy, GVD CC,
	LSS PRNG	SHA-512	BBI,	Color	Key-sensitivity, Histogram, PSNR, Occlusion,
		$2^{>310}$	2D-CA	(24 bit)	Chosen/known plain text, Variance.

**Table 7:** Histogram Variance Comparison.

Images	Plain			3C3R			Ref. [62]			Ref. [49]		
				Cipher			Cipher			Cipher		
	Red	Green	Blue	Red	Green	Blue	Red	Green	Blue	Red	Green	Blue
Lena	123,072.5	87,100.835	33,522.734	293.99	**264.977**	**254.722**	**247.78**	279.62	265.71	527.32	504.75	501.68
Couple	289,630.656	337,863.062	210,359.81	**242.434**	**216.323**	**246.357**	284.35	247.37	260.76	-	-	-
Female	113,045.289	64,436.410	66,971.062	298.134	**256.690**	243.771	**280.64**	280.46	**230.42**	-	-	-
Tree	129,825.531	57,011.605	81,373.710	**239.590**	**233.216**	238.281	282.81	254.87	**225.79**	-	-	-
Bean	168,076.796	501,640.093	789,945.75	**231.815**	**257.557**	**228.984**	232.98	279.61	245.61	-	-	-
House	992,034.12	1,330,180.12	768,126.75	**998.60**	**1107.34**	1046.96	1070.2	1231.2	**941.65**	-	-	-
All White	$2.67\times 10^{8}$	$2.67\times 10^{8}$	$2.67\times 10^{8}$	**220.130**	**203.561**	**216.067**	291.021	223.145	264.58	-	-	-

**Table 8:** Pixel correlation of 8k random pixel of Lena.

Algorithms		Cipher Image		
		Horizontal	Vertical	Diagonal
**3C3R**	**Plain**	0.95589	0.96567	0.93313
	**Cipher**	0.00750	**−0.00184**	**0.00012**
Ref. [15]		−0.0082	−0.0128	−0.0012
Ref. [66]		0.0020	−0.0009	**0.0017**
Ref. [28]		0.0265	0.0792	0.0625
Ref. [67]		0.0055	0.0041	0.002
Ref. [68]		**0.0005**	0.003	0.0021
Ref. [69]		0.0044	0.0034	0.0020
Ref. [70]		**0.0012**	0.0026	0.0021
Ref. [71]		0.0024	0.0012	0.0016
Ref. [72]		0.0072	0.0058	0.0031
Ref. [55]		0.0022	**0.0001**	**−0.0017**
Ref. [73]		0.0214	0.0465	−0.0090
Ref. [58]		−0.0077	**0.0002**	−0.0055

**Table 9:** Correlation Comparison of 1000 random Pixels of Lena.

Algorithm	Horizontal	Vertical	Diagonal	UACI	NPCR
Ref. [64]	0.003	−0.0040	**0.0013**	33.45	99.60
Ref. [63]	**0.0018**	0.0011	**−0.0013**	33.43	99.61
Ref. [74]	−0.0023	0.0019	−0.0034	33.51	99.62
Ref. [75]	**0.0020**	−0.0007	−0.0014	27.97	98.36
Ref. [65]	−0.0098	−0.0050	**−0.0013**	32.48	93.21
Ref. [76]	−0.0237	−0.0178	−0.0284	33.58	99.62
Ref. [77]	0.0080	0.0098	−0.0058	33.43	99.60
**3C3R**	−0.0027	**−0.00054**	**−0.0013**	**34.45**	**99.998**

**Table 10:** Pixel Correlation.

Images	Channels	Plain			Cipher			Entropy
		Horizontal	Vertical	Diagonal	Horizontal	Vertical	Diagonal	(R,G,B)
Lena.jpg	Red	0.95589	0.96567	0.93313	0.00750	−0.00184	0.00012	7.9972
	Green	0.93722	0.95832	0.92499	0.036575	0.002284	−0.003513	7.9974
	Blue	0.91142	0.93501	0.88513	0.0014717	−0.008797	0.0096045	7.9967
Baboon.png	Red	0.92283	0.86082	0.85468	−0.003068	0.004990	−0.002213	7.999
	Green	0.86721	0.76839	0.7416	0.0076227	−0.002984	−0.007508	7.999
	Blue	0.91092	0.88181	0.83619	0.012334	0.0007122	0.006166	7.9994
Fruits.jpg	Red	0.9865	0.98558	0.97342	−0.006591	−0.021533	−0.008414	7.996
	Green	0.98127	0.97943	0.96401	−0.003553	0.015646	−0.003781	7.9968
	Blue	0.95148	0.94673	0.91089	0.0085275	0.0084825	−0.002547	7.9972
Pepper.bmp	Red	0.96088	0.96686	0.95436	−0.001994	−0.007431	−0.009151	7.999
	Green	0.98276	0.98156	0.96989	−0.004029	−0.001068	−0.001439	7.9992
	Blue	0.967	0.96797	0.94546	0.0011452	0.00081563	−0.005897	7.9998
Skull.png	Red	0.98542	0.99226	0.97901	0.003727	0.007569	−0.001029	7.999
	Green	0.98546	0.99283	0.9822	−0.009794	0.017652	−0.001104	7.9993
	Blue	0.98659	0.99249	0.98009	−0.009525	−0.010168	−0.008972	7.999
Nike.png	Red	0.98823	0.99089	0.972	−0.0876	0.000835	−0.007701	7.999
	Green	0.98618	0.99008	0.9706	−0.095098	0.0008801	0.0003289	7.9997
	Blue	0.98723	0.9906	0.97178	−0.008857	0.003444	0.0054982	7.999
Playboy.png	Red	0.97007	0.98775	0.95954	−0.08556	−0.006046	−0.000411	7.999
	Green	0.97039	0.98257	0.95089	−0.10513	−0.001043	−0.000546	7.999
	Blue	0.96919	0.98127	0.95565	−0.002814	−0.005621	0.0066023	7.999
Airplane.bmp	Red	0.94741	0.93617	0.88936	−0.029442	−0.001137	−0.008049	7.9975
	Green	0.94242	0.94759	0.90503	−0.034386	−0.000201	−0.014404	7.9971
	Blue	0.9586	0.92611	0.90723	0.013706	−0.009122	−0.001553	7.9973
Bike.png	Red	0.95786	0.95752	0.92943	−0.003292	0.0047614	0.0073559	7.9992
	Green	0.96244	0.96579	0.935	0.013224	0.007460	−0.004485	7.999
	Blue	0.97765	0.97543	0.96262	−0.003638	−0.003146	0.0006025	7.9992
Opera.png	Red	0.97407	0.97118	0.9524	−0.000680	0.004448	−0.01049	7.999
	Green	0.96834	0.96442	0.94837	0.0009368	0.0007775	−0.004144	7.999
	Blue	0.97498	0.97311	0.95446	−0.001574	0.0000351	−0.01126	7.9993
Bridge.png	Red	0.95037	0.97699	0.92247	−0.004985	0.0036574	−0.002305	7.9993
	Green	0.95693	0.97946	0.92737	−0.000379	0.008972	0.003696	7.999
	Blue	0.96218	0.98441	0.94551	0.0069794	0.017072	−0.001523	7.9992
Vegetables.jpg	Red	0.97696	0.97952	0.96247	0.005099	−0.005643	−0.001603	7.999
	Green	0.97391	0.9767	0.95684	0.002007	0.009278	0.003720	7.999
	Blue	0.96823	0.96769	0.94104	−0.007227	−0.004639	−0.003687	7.999

**Table 11:** NPCR and UACI comparison.

Algorithm	Lena		Pepper	
	NPCR_R,G,B_	UACI_R,G,B_	NPCR_R,G,B_	UACI_R,G,B_
**3C3R**	**99.978**	33.46	**99.998**	**34.54**
Ref. [28]	99.66	33.44	99.63	33.47
Ref. [58]	99.599	33.465	-	-
Ref. [59]	99.62	**33.65**	-	-
Ref. [79]	99.62	**33.77**	99.64	33.53
Ref. [81]	**99.71**	33.45	**99.74**	**33.53**
Ref. [80]	99.60	33.48	-	-
Ref. [83]	99.6037	33.44	-	-
Ref. [84]	99.61	33.463	99.608	33.49

**Table 12:** NPCR and UACI values for special plaintexts.

**Images**	**NPCR_R,G,B(99.6174)_**			**UACI_R,G,B(33.4738)_**		
	**Red**	**Green**	**Blue**	**Red**	**Green**	**Blue**
Full Black	99.7021	99.6903	99.6905	33.4641	33.4412	33.4710
All White	99.7025	99.6912	99.6908	33.4715	33.4698	33.4708
SP_image1_	99.5975	99.4875	99.4764	33.4355	33.5970	33.4466
SP_image2_	99.6105	99.5091	99.5622	33.4344	33.5201	33.4649

**Table 13:** PSNR and MSE comparison under different cropping size.

Cropped Size	Proposed 3C3R		Ref. [29]	
	PSNR	MSE	PSNR	MSE
1/2	**12.88**	**3121.1**	11.58	4578.34
1/4	**14.722**	**2192.2**	14.59	2289.90
1/8	16.75	1375.9	**17.57**	**1155.32**
1/16	19.25	772.65	**20.57**	**579.98**

**Table 14:** PSNR between Plain (O) and cipher (C) image & Plain and Decrypted(D) image.

Algorithm	PSNR	Lena	Baboon	Couple	Panda	Vegetables
**3C3R**	O to D	*∞*	*∞*	*∞*	*∞*	*∞*
	O to C	**8.1020**	8.011	**6.2414**	**7.7028**	**6.8459**
Ref. [15]	O to C	8.1300	**7.8569**	7.4892	7.7410	7.4395
Ref. [46]	O to D	96.295	-	-	-	-
	O to C	9.0348	-	-	-	-
Ref. [23]	O to C	8.6878	-	-	-	-
Ref. [92]	O to C	9.0486	-	-	-	-
Ref. [90]	O to C	8.3655	8.8532	-	-	-
Ref. [91]	O to C	8.2522	8.8223	-	-	-

**Table 15:** Information Entropy Comparison.

Algorithm	Test	Plain			Ciphered		
	Image	R	G	B	R	G	B
	Lena	7.568	7.058	6.779	**7.997**	**7.997**	**7.996**
	Pepper	7.338	7.496	7.058	**7.999**	**7.999**	**7.999**
**Our**	Baboon	7.706	7.474	7.752	**7.999**	**7.999**	**7.999**
**3C3R**	Panda	7.708	7.552	7.726	**7.996**	**7.997**	**7.997**
	Vegetable	7.905	7.674	6.345	**7.999**	**7.999**	**7.999**
Ref. [82]	Lena	7.293	7.581	7.085	7.989	7.989	7.989
	Pepper	7.331	7.524	7.079	7.989	7.988	7.989
	Baboon	7.700	7.512	7.765	7.989	7.989	7.988
	Panda	7.711	7.627	7.793	7.988	7.989	7.989
	Vegetable	7.797	7.821	7.359	7.989	7.989	7.989
Ref. [87]	Lena	-	-	-	7.987	7.987	7.986
Ref. [88]	Lena	-	-	-	7.927	7.974	7.970
Ref. [89]	Lena	-	-	-	7.973	7.975	7.971
Ref. [96]	Lena	-	-	-	7.987	7.988	7.987

**Table 16:** Global Entropy and Shannon Entropy.

Images	Our 3C3R	${\mathrm{Global}}_{R,G,B}$		Ref. [95]
	Shannon_C_	Plain	Cipher	Shannon_C_
Vegetables	7.9028	7.496	7.999	-
Bridge	7.9023	7.876	7.999	-
Opera	7.9018	7.798	7.999	-
Bike	7.9023	7.441	7.999	-
Airplane	7.9027	6.665	7.9991	-
House	7.9027	7.4858	7.9998	7.9021
Playboy	7.9030	0.5257	8.00	-
Nike	7.9029	1.1969	7.9998	-
Skull	7.9019	7.483	7.999	-
Pepper	7.9029	7.669	7.999	7.9024
Fruit	7.9019	7.532	7.998	-
Baboon	7.9025	7.7624	7.9998	7.9023
Lena	7.9028	7.4517	7.9991	7.9024
All White	7.9020	0	8.00	-
Full Black	7.9027	0	8.00	-

**Table 17:** GVD Comparison.

USC-SIPI	Our 3C3R			Ref. [29]			Ref. [69]			Ref. [97]		
	GVD			GVD			GVD			GVD		
	Red	Green	Blue	Red	Green	Blue	Red	Green	Blue	Red	Green	Blue
4.1.01	**0.981**	**0.984**	0.923	0.977	0.979	**0.975**	-	-	-	-	-	-
4.1.02	**0.984**	**0.989**	**0.987**	0.978	0.979	0.979	-	-	-	-	-	
4.1.03	0.886	0.774	0.814	**0.978**	**0.976**	**0.977**	-	-	-	-	-	-
4.1.04	**0.988**	**0.978**	0.966	0.979	0.975	**0.980**	-	-	-	-	-	-
4.1.05	**0.983**	0.921	**0.975**	0.982	**0.966**	0.969	-	-	-	-	-	-
4.1.06	**0.986**	**0.998**	**0.976**	0.943	0.912	0.934	-	-	-	-	-	-
4.1.08	0.700	**0.979**	0.921	**0.985**	0.973	**0.983**	-	-	-	-	-	-
4.2.01	0.958	**0.998**	**0.986**	**0.989**	0.968	0.977	-	-	-	-	-	-
4.2.03	**0.986**	0.988	**0.992**	0.936	0.906	0.903	-	-	-	0.9801	**0.989**	0.9865
4.2.07	**0.995**	**0.953**	0.847	0.976	0.948	**0.974**	-	-	-	-	-	-
Lena	0.960	**0.9856**	**0.9874**	-	-	-	**0.9805**	0.9812	**0.9876**	0.9701	0.9700	0.9690

**Table 18:** Performance comparison.

Image Size	Proposed 3C3R	Ref. [98]	Ref. [81]	Ref. [99]	Ref. [57]
256×256	**3.321** s	4.7795 s	-	3.617 s	-
512×512	**6.713** s	8.670 s	8.308 s	14.811 s	16.170 s

**Table 19:** General Random terms Comparison With Some Most Recent Algorithms.

Algorithms	Images		Entropy Comparison		
			Red	Green	Blue
S.S Moafimadani^2019^ [100]	Full white		7.9994	7.9994	7.9993
	Full black		7.9993	7.9994	7.9993
Z. Liu^2019^ [51]	Full white		7.9914	7.9942	7.9856
	Full black		7.9965	7.9948	7.9955
**3C3R**	Full white		**8.00**	**8.00**	**8.00**
	Full black		**8.00**	**8.00**	**8.00**
M. Wang^2019^ [101]	Lena		7.9970	7.9973	**7.9973**
**3C3R**	Lena		**7.9972**	**7.9974**	7.9967
			**Histogram Variance**		
X. Chai^2019^ [62]	House		Red	Green	Blue
			1070.2	1231.2	**941.65**
**3C3R**	House		**998.60**	**1107.34**	1046.96
			**Correlation Comparison**		
W. Zhang^2019^ [102]	Pepper		Horizontal	Vertical	Diagonal
		Red	0.003853	**0.001284**	**−0.001832**
		Green	**−0.000912**	0.001460	0.002366
		Blue	−0.001647	0.006770	**−0.000366**
**3C3R**	Pepper	Red	**−0.001994**	−0.007431	−0.009151
		Green	−0.004029	**−0.001068**	**−0.001439**
		Blue	**0.0011452**	**0.00081563**	−0.005897
			**UACI Comparison**		
S.Suri^2019^ [56]	Lena		32.1752		
	Baboon		30.3547		
**3C3R**	Lena		**33.45**		
	Baboon		**33.43**		
			**NPCR, UACI Comparison**		
K.A.K Patro^2019^ [103]	Lena	NPCR	99.6314		
		UACI	**33.551**		
**3C3R**	Lena	NPCR	**99.978**		
		UACI	33.45		
			**Corr. Comparison Of 1000 pixels**		
P. Ramasamy^2019^ [76]	Lena		Horizontal	Vertical	Diagonal
			−0.0237	−0.0178	−0.0284
**3C3R**	Lena		**−0.0027**	**−0.00054**	**−0.0013**
			**PSNR Comparison**		
X. Liu^2019^ [91]	Lena	O to C	8.2522		
	Baboon	O to C	8.8223		
**3C3R**	Lena	O to C	**8.1020**		
	Baboon	O to C	**8.011**		
